# Comprehensive Investigation of the Caveolin 2 Gene: Resequencing and Association for Kidney Transplant Outcomes

**DOI:** 10.1371/journal.pone.0063358

**Published:** 2013-05-07

**Authors:** Jennifer A. McCaughan, Seamus Duffy, Thomas O'Hagan, Aisling E. Courtney, Richard Borrows, Peter J. Conlon, Alexander P. Maxwell, Amy Jayne McKnight

**Affiliations:** 1 Nephrology Research Group, Queen's University, Belfast, Northern Ireland; 2 Regional Nephrology Unit, Belfast City Hospital, Belfast, Northern Ireland; 3 Department of Nephrology and Transplantation, University Hospital Birmingham, Birmingham, United Kingdom; 4 On behalf of the Dublin Kidney Transplant Research Group, Renal Unit, Beaumont Hospital, Dublin, Ireland; University of Louisville, United States of America

## Abstract

Caveolae are plasma membrane structures formed from a complex of the proteins caveolin-1 and caveolin-2. Caveolae interact with pro-inflammatory cytokines and are dysregulated in fibrotic disease. Although caveolae are present infrequently in healthy kidneys, they are abundant during kidney injury. An association has been identified between a *CAV1* gene variant and long term kidney transplant survival. Chronic, gradual decline in transplant function is a persistent problem in kidney transplantation. The aetiology of this is diverse but fibrosis within the transplanted organ is the common end point. This study is the first to investigate the association of *CAV2* gene variants with kidney transplant outcomes. Genomic DNA from donors and recipients of 575 kidney transplants performed in Belfast was investigated for common variation in *CAV2* using a tag SNP approach. The *CAV2* SNP rs13221869 was nominally significant for kidney transplant failure. Validation was sought in an independent group of kidney transplant donors and recipients from Dublin, Ireland using a second genotyping technology. Due to the unexpected absence of rs13221869 from this cohort, the *CAV2* gene was resequenced. One novel SNP and a novel insertion/deletion in *CAV2* were identified; rs13221869 is located in a repetitive region and was not a true variant in resequenced populations. *CAV2* is a plausible candidate gene for association with kidney transplant outcomes given its proximity to *CAV1* and its role in attenuating fibrosis. This study does not support an association between *CAV2* variation and kidney transplant survival. Further analysis of *CAV2* should be undertaken with an awareness of the sequence complexities and genetic variants highlighted by this study.

## Introduction

Transplantation is the optimum treatment for end-stage kidney disease (ESKD). A kidney transplant improves the quality of life and survival of the recipient and substantially reduces the cost of ESKD to the healthcare provider [1], [2]. One year transplant survival rates following kidney transplantation have improved substantially in the last two decades; death censored transplant loss within the first 12 months has decreased from 15.7% in 1989 to 4% in 2008 [3]. However, the improvements in longer term kidney transplant survival have been less impressive [3], [4]. Chronic and gradual loss of kidney transplant function is due to myriad immunological and non-immunological insults. These include chronic antibody mediated rejection, calcineurin inhibitor toxicity, recurrent infection, urinary tract obstruction, hypertension and *de novo* or recurrent glomerular disease [5]–[7]. The cumulative injury to the transplant causes vascular and glomerular remodelling, extracellular matrix expansion, tubular atrophy and fibrogenesis [5], [7], [8]. Widespread fibrosis of the transplanted kidney is the final common endpoint [6].

Caveolae are flask-shaped, plasmalemmal invaginations formed from a stable hetero-oligomeric complex of the proteins caveolin 1 (CAV1) and caveolin 2 (CAV2) combined with cholesterol and sphingolipid rich molecules [9], [10]. Caveolae facilitate protein transcytosis, ion channel regulation, cholesterol transport and endocytosis of toxins, viruses and signalling molecules. These intricate structures are present in many cell types but are most abundant in adipocytes, endothelial cells, type 1 pneumocytes, myocytes, and fibroblasts [11].

Interaction exists between the caveolin binding domains and the high concentrations of signal transduction proteins contained within caveolae. Caveolar endocytosis and degradation of these proteins result in down-regulation of the signalling cascade [11], [12]. Transforming growth factor beta (TGFβ) is a pro-fibrotic cytokine which plays a key role in the initiation and propagation of fibrosis within the kidney [13]. The generation of pro-fibrotic proteins is up regulated by TGFβ with simultaneous loss of cell adhesion molecules leading to aberrant cell migration and compromise of the tubular basement membrane coupled with fibroblast proliferation and invasion [13]. Myofibroblasts differentiate from resident interstitial fibroblasts under TGFβ stimulation and TGFβ promotes calcineurin inhibitor-induced kidney transplant fibrosis [13], [14]. TGFβ receptors are contiguous with and located within caveolae; TGFβ is down regulated by caveolar endocytosis of this signalling molecule [12], [15]. CAV1 further suppresses TGFβ by interacting with the inhibitory Smad pathway causing TGFβ receptor degradation [9], [16]. CAV1 is recognised as an inhibitor of both cell proliferation and fibrosis and is known to be dysregulated in fibrotic diseases such as systemic sclerosis, pulmonary fibrosis, fibrosing cardiomyopathy, and keloid formation [15], [17], [18].

Healthy glomerular and peritubular capillary endothelial cells have few caveolae [19], [20]. However, in chronic antibody mediated rejection, substantial numbers of caveolae are found in endothelial cells and the degree of *CAV1* expression correlates with the pathological severity of rejection (graded by the Banff Score) [20], [21]. There is also abundant production of CAV1 in the glomerular endothelium of patients with glomerulonephritis [19]. In animal models of tubulointerstitial scarring, reduced *CAV1* expression is associated with increased tubulointerstitial injury and fibrosis [22], [23]. An association has recently been identified between a *CAV1* gene variant and kidney transplant survival. The donor *CAV1* single nucleotide polymorphism (SNP) rs4730751 is significantly associated with transplant failure and an increased incidence of transplant fibrosis [15]. In a replication cohort, this association with kidney transplant failure was demonstrated with both the donor and recipient *CAV1* SNPs [15].

In contrast to CAV1, little is known about the function of CAV2 although it has been implicated in type 2 diabetes mellitus [24], systemic sclerosis [18], cardiac conduction defects [25], cancer [26], [27], and primary open angle glaucoma [28]. Its role in the development of fibrosis has not been established. The *CAV2* gene is adjacent to the *CAV1* locus at 7q31.1 and is a plausible candidate gene for association with kidney transplant survival.

This study is the first to investigate the association between *CAV2* gene variants and kidney transplant outcomes. The primary end-point was death-censored transplant failure. Variants in the *CAV2* gene were genotyped for both donors and recipients of first deceased donor kidney transplants with validation sought in an independent cohort of kidney transplant donors and recipients from Dublin, Ireland. Both donor and recipient genomes may affect transplant outcomes since cells from each are implicated in vascular and glomerular remodelling during chronic transplant injury [29]–[31].

## Materials and Methods

### Ethics Statement

Ethical approval was granted for this study by the Office for Research Ethics Committees Northern Ireland (http://www.orecni.org.uk, 08/NIR3/79). Clinical follow up data has been stored in a regional transplant database since 1969. Written consent is obtained prospectively from recipients, or their guardians in the case of minors, for the storage of this data. However, written consent was not obtained from all recipients prior to 2006. The regional ethics committee waived the requirement for written consent from these recipients and granted permission for all of this data to be used in research involving the corresponding transplant DNA samples. All kidney transplant recipient and kidney donor data is fully anonymised by the clinical team prior to analysis. It is not possible for researchers to identify recipients from the data.

### Patient cohort

The Regional Nephrology Unit at Belfast City Hospital, Belfast is the only kidney transplant centre in Northern Ireland. Since 1986, genomic DNA has been obtained and stored from all recipient-donor pairs in first deceased donor kidney transplants at this centre.

There were 707 first, deceased donor transplants between May 1986 and April 2005. DNA was available for genotyping from 575 recipient-donor pairs. Clinical data and outcomes were recorded prospectively for all transplant recipients. Over 99% of both populations were White. Transplant failure is defined as a move to an alternative renal replacement modality such as dialysis therapy (death-censored transplant failure).

### Single Nucleotide Polymorphism selection for *CAV2*

**Figure 1:**
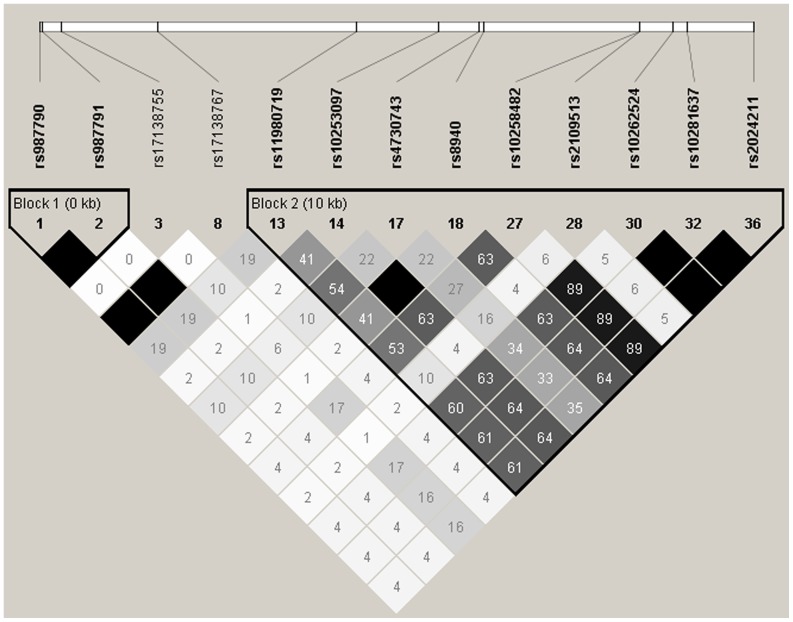
Haploview plot showing linkage disequilibrium between original tag SNPs downloaded from the International HapMap Project (release 28).

Genotype data for SNPs across the *CAV2* gene, including 5 kb flanking the *CAV2* 5′ and 3′ untranslated regions, was downloaded from the International HapMap Project [32], release 28 (http://hapmap.ncbi.nlm.nih.gov), for the CEPH population (Utah residents with ancestry from northern and western Europe). Information was available for 36 SNPs, of which 13 met the criteria of a minor allele frequency (MAF) >5%, Hardy Weinberg equilibrium > 0.001 and successful genotyping rate >95%. All SNPs had a MAF of at least 10%. Haploview [33] (version 4.2, www.broadinstitute.org/haploview) was used to identify linkage disequilibrium between SNPs and visualise haplotype blocks (Figure 1); seven tag SNPs were selected using a pairwise approach where r^2^>0.8. These seven tag SNPs {rs10258482, rs17138767, rs10253097, rs2109513, rs4730743, rs17138755 and rs11980719} are sufficient to examine all recorded common variation across the genetic region encompassing the *CAV2* gene.

The Ensembl Genome Browser (www.ensembl.org) was searched for putatively functional SNPs in *CAV2*; a further three non-synonymous SNPs for *CAV2* {rs13234554, rs13221869 and rs8940} were identified.

### Genotyping

Eight SNPs were genotyped using Sequenom iPLEX technology (Sequenom, Hamburg, Germany) and two using Taqman technology (Applied Biosystems, Warrington, Cheshire, UK). Recipient and donor DNA samples were randomly arranged in a 384-well format with four father-mother-proband trios and four negative controls per plate. The full details of primers, reaction conditions, equipment and software utilised are available from the authors.

### Power Calculation

Statistical power was calculated using StatCalc (version 6). This discovery cohort has >80% power to identify a risk allele of OR≥1.5 at the 5% significance level, assuming a MAF of 10%.

### Replication

SNPs that showed nominal association between SNP and transplant survival in the discovery cohort were genotyped in an independent cohort of 144 kidney transplant recipient-donor pairs from Beaumont Hospital, Dublin, Ireland. These donor and recipient cohorts are comparable to the Belfast populations in terms of ethnicity, age and primary kidney disease. Taqman technology was used for replication genotyping.

### Direct Capillary Sequencing

**Figure 2:**
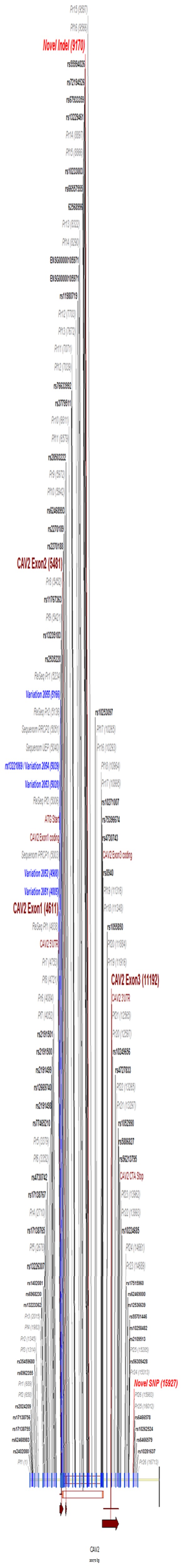
Schematic diagram of *CAV2*.

**Figure 3:**
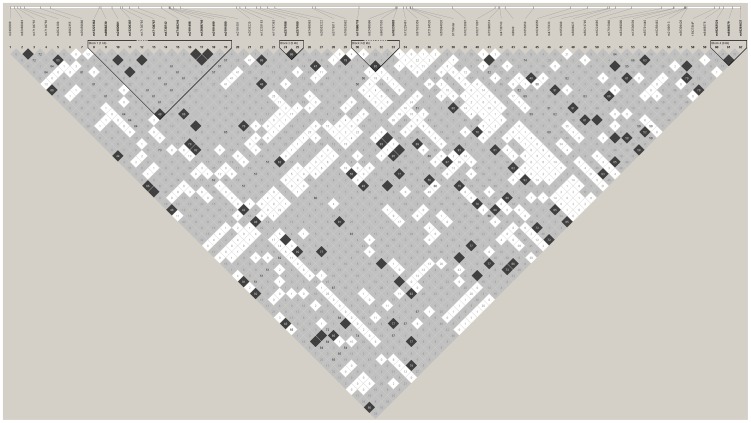
Haploview plot showing linkage disequilibrium between *CAV2* SNPs using resequencing data.

The *CAV2* reference sequence (homo sapiens chr7 (GRCh37:115924434...116151595)) was obtained from GenBank at the National Center for Biotechnology Information (www.ncbi.nlm.nih.gov/genbank/). The genomic region of interest was extended to 4.5 kb upstream from the reference mRNA transcription start site and 6 kb downstream from the stop codon to also investigate functional regions that may influence expression of *CAV2*. Twenty-six overlapping fragments were PCR amplified using genomic DNA from 31 individuals. The average fragment length was 692 base pairs. Bidirectional sequencing was undertaken using Genetic Analyser 3730 (Applied Biosystems, Warrington, UK) and sequencing conditions are available from the authors. Contigs were mapped to the reference genome and visualised using Vector NTI (Invitrogen Ltd, Paisley, UK) (Figure 2). Haploview [33] was used to identify linkage disequilibrium between SNPs and visualise haplotype blocks (Figure 3).

### Statistical Analysis

Genotype distributions for recipient and donor groups were assessed for Hardy-Weinberg equilibrium. Cumulative transplant and recipient survival were analysed using Kaplan-Meier survival plots with the log rank test assessing inter-group variation.

There are a number of clinical features including donor age, recipient age, recipient gender, diabetic nephropathy, acute rejection, decade of transplantation, ischemic time and degree of HLA mismatching which are generally accepted to impact transplant and/or recipient survival. There were also significant improvements in transplantation during this period. To allow for this in the analysis, each transplant was coded according to the decade of transplantation; decade one encompassed transplants performed between 1986 and 1995 and decade two, those performed between 1996 and 2005 inclusive. Log rank testing was used to assess the impact of these variables on transplant outcome. A Cox proportional-hazards model was used to perform multiple regression analysis incorporating variables which impact transplant survival.

Genotype and allelic group comparisons were made using the Chi-squared test.

A p value of <0.05 was considered nominally statistically significant in all analyses. SPSS for Windows (SPSS*®* Inc., Chicago, Illinois) version 17.0 was used for all analyses.

## Results

### Clinical

There were 707 first deceased donor kidney transplants during the period studied. The average age of recipients was 42 years (range 2–77 years) and the average age of kidney donors was 37 years (range 1–75 years). 439 (62.1%) of recipients and 428 (60.5%) of donors were male (Table 1).

**Table 1:** Clinical characteristics of Belfast Kidney Transplant Population.

Variable	n
Recipient age/years	
Mean (SD)	42 (16.7)
Recipient gender	
Male	439 (62%)
Donor age/years	
Mean (SD)	37 (16.8)
Donor gender	
Male	428 (61%)
Primary renal disease	
Glomerulonephritis	139 (20%)
Intersitial/pyelonephritis	144 (20%)
Autosomal Dominant Polycystic Kidney Disease	103 (15%)
Diabetic nephropathy	71 (10%)
Other	157 (22%)
Unknown	93 (13%)
Decade of transplantation	
1986–1995	380 (54%)
1996–2005	327 (46%)
HLA mismatch/number of mismatches	
Mean (SD)	2.2 (1.1)
Ischaemic time/minutes	
Mean (SD)	1428 (440)
Acute rejection*	143 (20%)

* Biopsy proven within 6 months of transplant

The median follow up time in this study was 12.2 years (range 0–26.0 years). There were 438 transplant failures: 187 recipients died with a functioning transplant and 251 transferred to an alternative mode of renal replacement therapy. There were 105 deaths in the latter group within the follow up period.

Donor age (p<0.001), acute rejection within 6 months (p<0.001) and earlier decade of transplantation (p = 0.026) were significantly associated with transplant failure. Recipient age (p<0.001), donor age (p = 0.002), diabetic nephropathy as the primary renal diagnosis (p<0.001), and earlier decade of transplantation (p = 0.005) were significantly associated with recipient mortality. For the purpose of this analysis, donor and recipient age were grouped into decades. The degree of HLA mismatching across A, B and DR loci did not significantly influence transplant outcomes. As has previously been described, this probably reflects the policy of favourable matching at this centre. Only 1% of this cohort had two mismatches at the DR locus [34], [35].

### Genotyping

**Figure 4:**
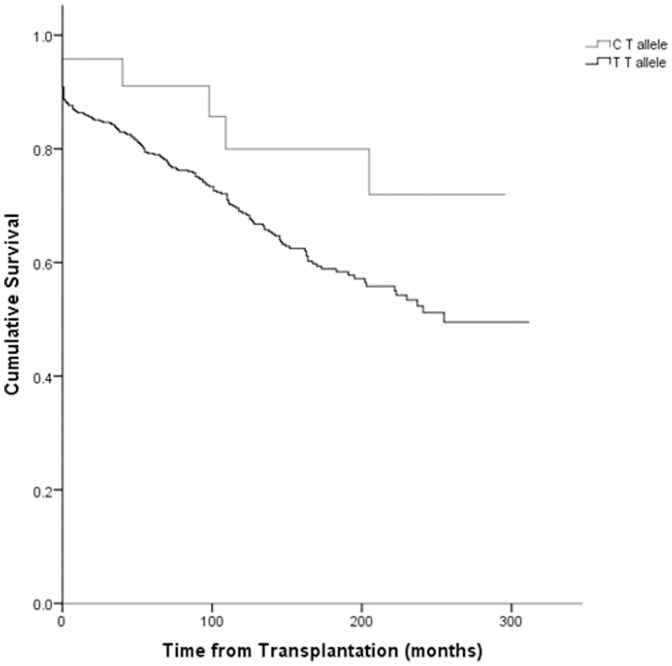
Kaplan-Maier plot showing association between recipient *CAV2* SNP rs13221869 and transplant survival.

The average genotyping success rate was 93%. Genotypes for SNP rs4730743 and rs10258482 deviated from Hardy-Weinberg Equilibrium in both recipients and controls and were therefore excluded from further analysis. SNP rs13234554 was also excluded due to unreliable genotype calls. Recipient genotyping data and associations with transplant survival are shown in Table 2. There was no association between donor variability and transplant outcomes. Using Kaplan-Meier survival analysis, significant associations were identified between transplant survival and recipient variants at the tag SNP rs11980719 (p = 0.024). The most significant association with a non-synonymous SNP was with recipient rs13221869 (p = 0.085). The presence of a T allele was protective for rs11980719 and the presence of a C allele was associated with transplant survival benefit for rs13221869 (Figure 4). There were no donors or recipients who were homozygous for a C allele at rs13221869. Analysis revealed that rs11980719 and rs13221869 are not in strong linkage disequilibrium (r^2^ 0.635).

**Table 2:** Genotype data and association with transplant survival for *CAV2* SNPs.

SNP	Genotype	Recipient	p value	Donor	p value
8940	CC/CG/GG	369/132/15	0.831	378/165/21	0.608
10253097	TT/TC/CC	372/134/18	0.208	360/138/20	0.890
**11980719**	**TT/TA/AA**	**237/241/72**	**0.024**	**216/270/73**	**0.998**
**13221869**	**TT/TC/CC**	**504/24/0**	**0.085**	**516/23/0**	**0.470**
17138755	AA/AC/CC	439/99/8	0.326	440/101/7	0.558
17138767	AA/AG/GG	461/73/3	0.304	353/79/4	0.962
2109513	CC/CT/TT	410/143/11	0.097	402/169/13	0.663

There was no significant association between rs11980719 (p = 0.375) or rs13221869 (p = 0.926) and biopsy proven acute rejection within the first 6 months of transplantation. Insufficient transplant biopsy results are available to identify any correlation between *CAV2* SNPs and a specific aetiology of chronic transplant failure. There was no significant association between these SNPs and recipient survival.

A Cox regression-proportional hazards model was used to correct for variables which were significantly associated with transplant survival in our population (donor age, acute rejection within 6 months and decade of transplantation) as well as those which are generally accepted to impact transplant survival but that did not reach statistical significance in this cohort (recipient gender, recipient age, degree of HLA mismatching, ischemic time). In this analysis, the association between rs13221869 and transplant survival was magnified (HR 0.422, CI 0.173–1.031, p = 0.058) (Table 3) but this was not the case with rs11980719 (HR 1.295, CI 0.872–1.922, p = 0.20).

**Table 3:** Cox regression analysis of transplant survival.

Variable	Hazard ratio	Confidence Intervals	p value
Donor age (per decade)	1.200	1.091–1.319	0.000
Decade of transplantation	0.772	0.564–1.057	0.107
Acute rejection °	2.015	1.469–2.765	0.000
Recipient gender	0.949	0.701–1.285	0.735
Recipient age (per decade)	0.854	0.776–0.939	0.001
HLA match	1.031	0.889–1.195	0.688
Ischaemic time (tertiles)	0.864	0.686–1.089	0.217
**rs13221869**	**0.422**	**0.173–1.031**	**0.058**

° Biopsy proven acute rejection within 6 months of transplant

### Replication

When rs13221869 was genotyped in the replication cohort, the SNP was unexpectedly reported as monomorphic. A second set of Taqman primers and probes were designed but genotyping once again detected a single allele at this locus. The Belfast and Dublin transplant populations are genetically similar and the failure to identify rs13221869 in this population was surprising. This, along with the unusually high proportion of SNPs that failed quality control, led us to resequence this potentially important biological and positional candidate gene.

### Direct Capillary Sequencing

17 kb surrounding the *CAV2* gene was bi-directionally resequenced. There were 181 unique SNPs reported in the *CAV2* gene; 60 of these were confirmed in our population (Table 4, Figure 2). Six of these known variations deviated from Hardy Weinberg equilibrium in the resequencing study; the SNPs rs55994026, rs67933359, rs2024209 and rs13229461 and two insertion/deletions rs35459680 and rs72194526. In addition, one novel insertion/deletion (base position 9170, GAGG/-, ss550827879) and one novel SNP (base position 15927, A/T, ss550827880) were identified. Linkage disequilibrium between the SNPs and haplotype blocks is shown in Figure 3.

**Table 4:** SNPs, insertions and deletions identified in *CAV2* sequencing.

SNP	Base position	Base change	Genotype	Frequency	MAF
rs2402080	183	G>C	GG/GC/CC	14/11/6	0.371
rs62468983	290	G>A	GG/GA/AA	25/6/0	0.097
rs17138755	355	A>C	AA/AC/CC	25/6/0	0.097
rs17138756	420	A>G	AA/AG/GG	24/6/1	0.129
rs2024209	503	T>C	TT/TC/CC	27/0/3	0.100
rs6962355	859	G>A	GG/GA/AA	23/7/0	0.117
rs35459680	1088	G>-	GG/G-/--	28/0/2	0.483
rs13223362	2173	G>A	GG/GA/AA	11/12/6	0.414
rs6968230	2216	G>T	GG/GT/TT	28/1/2	0.081
rs2402081	2462	C>T	CC/CT/TT	1/6/23	0.129
rs13226307	2652	A>T	AA/AT/TT	28/1/2	0.081
rs17138765	2693	G>A	GG/GA/AA	29/2/0	0.032
rs17138767	2844	A>G	AA/AG/GG	29/1/1	0.081
rs4730742	3059	T>G	TT/TG/GG	18/10/3	0.258
rs77465210	3572	T>A	TT/TA/AA	26/3/1	0.081
rs2191498	3736	C>T	CC/CT/TT	27/1/2	0.083
rs12669740	3743	T>C	TT/TC/CC	27/1/2	0.083
rs2191499	3766	C>T	CC/CT/TT	27/1/2	0.083
rs2191500	3833	C>A	CC/CA/AA	8/10/12	0.433
rs2191501	3998	G>T	GG/GT/TT	13/1/1	0.048
rs2535220	5243	T>C	TT/TC/CC	1/7/16	0.188
rs13235183	5316	G>T	GG/GT/TT	22/1/1	0.062
rs11767363	5442	T>C	TT/TC/CC	1/6/8	0.129
rs2270188	5691	G>T	GG/GT/TT	14/11/5	0.350
rs2270189	5783	A>G	AA/AG/GG	13/11/6	0.383
rs62468993	5931	G>A	GG/GA/AA	27/3/0	0.050
rs28503222	6098	G>C	GG/GC/CC	16/9/4	0.293
rs3779511	6945	T>G	TT/TG/GG	9/16/6	0.452
rs76633992	6998	G>T	GG/GT/TT	13/7/11	0.468
rs11980719	7975	T>A	TT/TA/AA	9/16/4	0.414
rs62468996	8804	C>A	CC/CA/AA	28/1/0	0.017
rs66557555	8823	G>-	GG/G-/--	27/2/0	0.048
rs10233003	8829	C>A	CC/CA/AA	11/14/4	0.379
rs13229461	8969	C>T	CC/CT/TT	23/3/5	0.293
rs67933359	9029	C>A	CC/CA/AA	26/0/5	0.172
rs72194526	9117	Deletion	GAGGGAGG/-	21/9	0.310
rs55994026	9132	G>A	GG/GA/AA	23/0/8	0.241
**ss550827879**	**9170**	**GAGG>-**	**GAGG/-**	**30/1**	**0.016**
rs10253097	10071	T>C	TT/TC/CC	12/5/7	0.396
rs10271007	11016	A>G	AA/AG/GG	10/10/5	0.400
rs75396674	11104	T>A	TT/TA/AA	23/4/3	0.155
rs4730743	11124	A>T	AA/AT/TT	13/11/5	0.362
rs8940*	11241	C>G	CC/CG/GG	17/9/3	0.259
rs1055850*	11878	A>G	AA/AG/GG	1/8/22	0.161
rs10249656*	12704	C>T	CC/CT/TT	17/10/3	0.267
rs4727833*	13075	C>G	CC/CG/GG	19/7/5	0.117
rs1052990*	13537	T>G	TT/TG/GG	9/16/6	0.452
rs5886827*	13575	A>-	AA/A-/--	14/5/12	0.387
rs56213795	13711	G>A	GG/GA/AA	29/2/0	0.016
rs10224685	14067	A>C	AA/AC/CC	18/9/3	0.250
rs17515960	14743	A>G	AA/AG/GG	25/6/0	0.097
rs62469000	15011	C>T	CC/CT/TT	24/7/0	0.113
rs12536639	15012	G>A	GG/GA/AA	18/10/3	0.258
rs55701446	15244	G>T	GG/GT/TT	22/8/1	0.161
rs10258482	15262	C>A	CC/CA/AA	12/16/3	0.355
rs2109513	15275	T>C	TT/TC/CC	1/8/22	0.161
**ss550827880**	**15927**	**A>T**	**AA/AT/TT**	**27/4/0**	**0.048**
rs56309428	15304	G>T	GG/GT/TT	29/1/1	0.065
rs6466578	16036	C>T	CC/CT/TT	1/8/22	0.161
rs10262524	16119	C>A	CC/CA/AA	12/15/4	0.371
rs6466579	16322	T>C	TT/TC/CC	13/12/6	0.387
rs10281637	16505	T>C	TT/TC/CC	12/15/4	0.371

MAF – minimum allele frequency

* SNPs previously confirmed in a European population with MAF>5%

The primers initially designed to screen fragment 8 (750bp, Figure 2), which included rs13221869, failed at sequencing. Two further sets of sequencing primers were designed and a nested PCR was required to optimise the sequence data for this technically challenging region. Six individuals who were clearly heterozygous (T/C) for rs13221869 by Sequenom and two homozygotes (T/T) were resequenced. None of the reported SNPs in this region (variation 2051–2055) were present in our resequencing dataset. In samples which had been reported as rs13221869 (Variation 2054) heterozygotes using Sequenom, there was no evidence of single nucleotide variation at this locus. Detailed review of the failed sequencing data suggested that the initial sequenced DNA strands were misaligned by three bases. This led to the appearance of SNPs in reasonable sequencing chromatograms that were not present when the region was ‘correctly’ aligned using a long, clean sequencing read. Despite BLAST analysis at the Sequenom primer design stage and good predicted quality scores, there are multiple loci in the annealing sequence of the Sequenom unextended primer where a short identical sequence is repeated three bases upstream.

## Discussion

Improving long term outcomes in kidney transplantation remains a challenge. Transplant failure within the first year has been reduced by the development of new immunosuppressive drugs and advanced surgical techniques and a lasting survival benefit was expected [7], [30], [36]. The reality has been disappointing. While the rate of transplant loss within five years has significantly improved in the modern era, the long term attrition rate has remained largely unchanged [3], [4], [37].

This study follows the report of a risk variant in the *CAV1* gene that associates with poor transplant survival [15] and is the first to investigate the effect of variation in the *CAV2* gene on kidney transplant outcomes. SNPs at the *CAV2* locus were investigated and a trend towards association was suggested between rs13221869 in the recipient genome and transplant survival. There was no association with recipient survival or acute rejection. An attempt to replicate this association in an ethnically similar kidney transplant recipient-donor population using an alternative technology failed to genotype two alleles for this SNP.

Direct capillary sequencing was subsequently employed to investigate the *CAV2* gene in detail. Initial attempts to sequence the fragment encompassing rs13221869 failed (base position 5039, Pf8, Pr8, Figure 2) and an unusual degree of optimisation was required. This study highlights not only the need for validation of interesting SNP associations using an alternative technology, but also that future genetic studies of *CAV2* in particular warrant careful consideration. For example, our original Sequenom data and 3730 sequencing showed a C/T variant at base position 5039, which is the reported position of rs13221869. However, the use of a longer sequencing fragment that reads through the difficult region revealed this ‘SNP’ was due to misalignment of bases. This SNP (rs13221869) was originally identified via large-scale sequence comparisons and has not been confirmed by genotyping or population frequency data (www.ncbi.nlm.nih.gov/projects/SNP/snp_ref.cgi?rs=13221869).

Repetitive DNA sequences account for 50–80% of the human genome [38], [39]. The repetition of DNA sequences causes ambiguities in alignment and genome assembly in DNA sequencing and poses a significant problem. This is magnified by next generation sequencing technologies as a result of the formation of shorter DNA fragments where fewer bases are present to verify the corresponding position in the reference genome [38]. Uniform heterozygosity or excess heterozygosity resulting in Hardy Weinberg disequilibrium may suggest that a SNP has been identified as a result of sequence-read misalignment [40]. Misalignment of sequenced DNA resulted in the erroneous identification of the heterozygote SNP rs13221869, which appeared to be in Hardy Weinberg equilibrium when genotyped using Sequenom in this study.

The steps necessary for the discovery of a new genetic variant ought to be threefold: firstly, the detection of the variant (often by large scale, high throughput approaches); secondly, the validation of this finding in an independent population and thirdly, characterization of the variant using an alternative technology [41]. The National Center for Biotechnology Information *CAV2* SNP genotype report lists 86 known SNPs in the *CAV2* gene (accessed 06.11.2012). Of these, only 20 have been confirmed by population frequency or genotyping data, including two which were confirmed in populations of less than five individuals. This study identified six of the seven SNPs (rs8940, rs1052990, rs1055850, rs4727833, rs5886827, rs10249656, rs56213795) which have been validated in a population of European origin with a MAF>5% and provides population frequency data for an additional 54 SNPs. The Haploview plot of resequenced data illustrates the paucity of linkage disequilibrium between the confirmed SNPs (Figure 3). For this reason, it is not feasible to accurately assess variation within the *CAV2* gene using tag SNPs derived from the existing version of the HapMap project (Figure 1).

At the turn of the millennium, the cost of sequencing the human genome was $100,000,000 [42]. Today, the human genome may be sequenced in its entirety for less than $1,000 [43]. This rapid reduction in the cost of DNA sequencing and the exponential increase in the output of sequencing platforms have resulted in an unprecedented amount of information about the genetic code becoming available. This, however, must be matched by accurate and reproducible bioinformatics platforms for analysis and, even more importantly, careful interpretation of the results. This investigation of *CAV2* illustrates the importance of replication and detailed validation of findings in clinical genetic research.

Analysis of the human genome has provided useful insights into the pathogenesis of chronic kidney disease and kidney transplant outcomes [34], [44]–[46]. These insights have resulted in advances which are beginning to be translated into clinical practice [46]. *CAV2* is a plausible candidate gene for association with kidney transplant survival because of its proximity to the *CAV1* locus and its modulatory role in fibrosis and angiogenesis which are key pathological components of chronic transplant dysfunction [17], [29]. The advantage of employing different technologies in the investigation of *CAV2* is emphasised by this study.

This study did not identify a significant association between single nucleotide polymorphisms in *CAV2* and kidney transplant outcomes. However, it did identify novel variants, provide frequency data for known variants and provide a plausible explanation as to how a functional SNP might have been mistakenly identified and reported.

## Conclusion

This study is the first to investigate the role of recipient and donor *CAV2* variants in kidney transplant survival. There was no association between genetic variation at *CAV2* and either kidney transplant or recipient survival. However, the resequencing data identified novel SNPs, provided population data, and highlights the challenges inherent in genotyping *CAV2* variants. This study also demonstrates the necessity of ensuring correct sequence alignment and confirmation of variants from high throughput sequencing to ensure the validity of results.
